# Endogenous incretin levels and risk of first incident cancer: a prospective cohort study

**DOI:** 10.1038/s41598-023-27509-3

**Published:** 2023-01-07

**Authors:** Amra Jujić, Christopher Godina, Mattias Belting, Olle Melander, Jens Juul Holst, Emma Ahlqvist, Maria F. Gomez, Peter M. Nilsson, Helena Jernström, Martin Magnusson

**Affiliations:** 1 Department of Clinical Sciences Malmö, Lund University, Malmö, Sweden; 2 Department of Cardiology, Skåne University Hospital, Malmö, Sweden; 3 Department of Clinical Sciences, Lund University Diabetes Centre, Lund University, Malmö, Sweden; 4 Department of Clinical Sciences Lund, Oncology, Lund University and Skåne University Hospital, Lund, Sweden; 5 Department of Immunology, Genetics, and Pathology, Science for Life Laboratory, Uppsala University, Uppsala, Sweden; 6 Department of Biomedical Sciences and NNF Center for Basal Metabolic Research, The Panum Institute, Copenhagen, Denmark; 7 NNF Center for Basal Metabolic Research, University of Copenhagen, Copenhagen, Denmark; 8 Wallenberg Center for Molecular Medicine, Lund University, Malmö, Sweden; 9 Hypertension in Africa Research Team (HART), Northwest University Potchefstroom, Potchefstroom, South Africa; 10 Clinical Research Centre, Lund University, Box 50332, 202 13 Malmö, Sweden

**Keywords:** Cancer, Endocrinology

## Abstract

Concerns have been raised regarding a potentially increased risk of cancer associated with treatment with glucagon-like peptide-1 (GLP-1) receptor agonists. Here, we explored whether fasting and oral glucose tolerance test post-challenge glucose-dependent insulinotropic peptide (GIP) and GLP-1 levels were associated with incident first cancer. Within the cardiovascular re-examination arm of the population-based Malmö Diet Cancer study (n = 3734), 685 participants with a previous cancer diagnosis were excluded, resulting in 3049 participants (mean age 72.2 ± 5.6 years, 59.5% women), of whom 485 were diagnosed with incident first cancer (median follow-up time 9.9 years). Multivariable Cox-regression and competing risk regression (death as competing risk) were used to explore associations between incretin levels and incident first cancer. Higher levels of fasting GLP-1 (462 incident first cancer cases/2417 controls) showed lower risk of incident first cancer in competing risk regression (sub-hazard ratio 0.90; 95% confidence interval 0.82–0.99; p = 0.022). No association was seen for fasting GIP, post-challenge GIP, or post-challenge GLP-1 and incident first cancer. In this prospective study, none of the fasting and post-challenge levels of GIP and GLP-1 were associated with higher risk of incident first cancer; by contrast, higher levels of fasting GLP-1 were associated with lower risk of incident first cancer.

## Introduction

Following nutrient intake, incretin hormones (glucose-dependent insulinotropic polypeptide (GIP) and glucagon-like peptide-1 (GLP-1)) enhance glucose-dependent insulin response, resulting in a lowering of blood glucose^1^. Patients with diabetes suffer from impaired incretin effects. During the last two decades, incretin-based therapies (glucagon-like peptide 1 receptor agonists (GLP-1 RA) and dipeptidyl peptidase inhibitors (DPP-4i)) have provided safe and effective treatment options for patients with diabetes, along with beneficial effects such as weight loss^2^. In addition, several studies support therapeutic benefits of GLP-1 RA with regard to cardiovascular outcomes in type 2 diabetes^3^. However, as incretins stimulate cellular proliferation, concerns have been raised regarding a potentially increased risk of thyroid and pancreatic cancer, associated with GLP-1 RA usage^4–6^. Studies have reported increased risk of pancreatic cancer associated with GLP-1 RA usage^7–9^, while other studies did not find such an association^10,11^. The observed associations are often attributed to an occult pancreatic cancer that provokes or aggravates diabetes^7^. Considering the relatively short follow-up time in these studies, we hypothesize that it is likely that the mechanism is promotion of existing lesions, through the presence of supra-physiological levels of GLP-1, rather than induction of new tumors. Additionally, the risk of pancreatic cancer is increased in type 2 diabetes, further complicating the interplay between diabetes and cancer^12^. Further, Ligumsky et al. identified GLP-1 as a potent inducer of cyclic adenosine monophosphate and an inhibitor of breast cancer cell proliferation, suggesting that reduced GLP-1 levels may serve as a novel link between obesity, diabetes mellitus, and breast cancer^13^. Globally, cancer constitutes a huge burden on society and accounts for a significant proportion of deaths^14^. Substantial progress has been made during the last three decades in diagnosis and treatment of numerous cancers, while prevention is still behind the progress seen in relation to cardiovascular disease^14^. Pancreatic cancer remains a clinical challenge, especially regarding early diagnosis and surveillance^15^. There is an ongoing debate about the potential involvement of incretins in pancreatic cancer initiation and progression. To date, no reliable data exist on the association between endogenous levels of incretins and risk of cancer. Herein, we explored whether endogenous circulating GIP and GLP-1 levels are associated with incident first cancer(s) in the population-based Malmö Diet and Cancer Study.

## Methods

### Participants

Between 1991 and 1996, baseline examinations including anthropometrical measurements and blood sample donations were performed as part of the Malmö Diet and Cancer Study (MDC), a prospective population-based study (n = 30,447) in the city of Malmö, Sweden. A sample of the study population (n = 6103) was randomized into a sub-study “The Malmö Diet and Cancer Cardiovascular Cohort” (MDC-CC). During 2007–2012, a clinical re-examination including blood sampling was performed in 3734 participants within the MDC-CC cohort, with the addition of oral glucose tolerance test (OGTT) in participants without any diabetes diagnosis and fasting plasma glucose levels ≤ 7 mmol/L. Data from this re-examination was used for analyses. A complete description of the study population has been published elsewhere^16^. This study was performed in line with the principles of the Declaration of Helsinki. Approval was granted by the Research Ethical Review Board at Lund University. A written informed consent was obtained from all participants prior to commencement of the study.

### Clinical assessment

Clinical assessment included anthropometric measurements and blood samples drawn after overnight fast. Body mass index (BMI) was calculated as weight in kilograms divided by the square of the height in meters. Waist circumference (cm) was measured between the lowest rib margin and the iliac crest. Diabetes mellitus was identified through a self-reported physician’s diagnosis of diabetes, use of anti-diabetic medications, or by oral glucose tolerance testing (OGTT; fasting plasma glucose (FPG) ≥ 7.0 mmol/L or ≥ 12.2 mmol/L following OGTT). Smoking was self-reported and defined as present smoker/non-smoker. Use of menopausal hormone therapy (MHT) was self-reported and defined as never/ever/present. Insulin resistance was estimated by Homeostatic Model Assessment for Insulin Resistance (HOMA-IR)^17^. Physical activity was self-reported via questionnaire, concerned average activity during spare time in the last year, and defined as (1) *Sedentary* Leisure time primarily devoted to reading, needlework, TV, cinema or other sedentary activities; (2) *moderate exercise* Leisure time primarily devoted to walking, cycling or exercising at least 4 h per week; (3) *regular exercise* Leisure time primarily devoted to running, swimming, tennis, badminton, fitness exercises, or similar at least 3 h per week; and (4) *strenuous exercise* Leisure time primarily devoted to strenuous exercise and competition in running, orienteering, skiing, swimming, football, handball and the like, regularly and at least 4 times per week.

### Oral glucose tolerance testing

A standardized 75 g OGTT^18^ was performed after an overnight fast. Individuals with known diabetes and individuals with fasting plasma glucose ≥ 7 mmol/L did not undergo the OGTT and subsequently no post-challenge blood sampling. During OGTT, blood samples were drawn at 0 and 120 min for plasma glucose, serum insulin, glucagon, GIP, and GLP-1 analyses (2007–2012). Blood samples for GIP analyses were collected in serum tubes with a clot activator and serum gel separator and stored at − 20 °C until analyses (carried out November 2011 and June 2012). Blood samples for GLP-1 were collected in tubes with addition of a DPP-4i (Diprotin A) and stored at − 80 °C until analyses (during 2014).

### Laboratory assays

Total plasma GLP-1 concentrations (intact GLP-1 and the metabolite GLP-1 9–36 amide) were determined radio immunologically as described previously^19^ (minimum detection limit 1 pmol/L; intra- and inter-assay coefficients of variation < 6.0% and < 15%, respectively). Identical quality controls and identical batches for all reagents in each analysis set were used in a consecutive sample analysis for two months. Serum GIP was analyzed using Millipore’s Human GIP Total ELISA #EZHGIP-54K (total, minimum detection level 1.65 pmol/L, intra- and inter-assay coefficients of variation were 1.8–6.1%, and 3–8.8% respectively). FPG was analyzed after an overnight fast using the Hemocue Glucose System (HemoCue AB, Ängelholm, Sweden). Serum insulin was assayed with Dako ELISA kit (minimum detection level 3 pmol/L, intra- and inter-assay coefficients of variation 5.1–7.5% and 4.2–9.3% respectively) at the Department of Clinical Chemistry, Malmö University Hospital. Plasma glucagon was assayed with RIA GL-32 K (Merck Millipore, Dermstadt, Germany, minimum detection level 18.5 pg/mL, intra- and interassay coefficients of variation 3.6–6.2% and 8.7–14.7% respectively).

### Endpoints

Data on cancer diagnoses were identified by record linkage with regional and national cancer registries in Sweden, which have high coverage and accuracy of diagnosis^20^. Participants were followed from study entry (MDC-CC re-examination; 2007–2012) until first cancer diagnosis (other than nonmelanoma skin cancer and cervical cancer in situ) or otherwise censored (emigration, death or end of follow-up period 31 December 2019) using ICD7, ICD9 and ICD10 codes.

### Statistics

Non-normally distributed variables were ln-transformed (fasting and post-OGTT challenge incretins, glucose, insulin, glucagon, and HOMA-IR) and thereafter z-transformed. Correlations were explored using Spearman correlations. Linearity assumptions for variables in the Cox regression models examining associations between incretins and incident first cancer were checked by plotting Martingale residuals. Schoenfeld’s residuals were used to assess whether the proportional hazard assumption was violated for fasting and post challenge incretins and factors of metabolic health in *Model 2a* for the following endpoints: all incident cancers, hormone- and obesity-related cancers. The hazards were not proportional for fasting GLP-1 in relation to incident obesity-related cancers (*p* = 0.016). Subsequently, an interaction with time was included, both as a continuous variable and with a cut-off > 4 years. There were no other violations of the proportional hazard assumption.

### Analyses of associations between fasting concentrations and any incident first cancer

Prior to any analyses, all prevalent cases of cancer other than nonmelanoma skin cancer and cervical cancer in situ by the time of MDC-CC re-examination were excluded (n = 685), resulting in 3049 participants, among whom there were 485 incident cases of first cancer within a maximum follow-up period of 12.6 years (median 9.9 years, interquartile range (IQR) 8.6–11.4 years). For associations between fasting and post-challenge (1) GIP, (2) GLP-1, (3) glucose, (4) insulin and (5) glucagon with incident first cancer, participants with missing data for any variable in the following analyses were excluded. Cox regression models were used to obtain Hazard Ratios (HR) with 95% confidence intervals (95%CI), and significant findings were further analyzed in model (1) adjusted for age and sex (*Model 1*); and (2) further adjusted for waist circumference, diabetes, smoking status and physical activity (*Model 2a*). Sensitivity analyses of associations between concentrations of fasting and post-challenge (1) GIP, (2) GLP-1, (3) glucose, (4) insulin and (5) glucagon and any first cancer were carried out after exclusion of 297 participants with prevalent diabetes using Cox-regression models (1) adjusted for age and sex (*Model 1*); further adjusted for waist circumference, smoking status, physical activity, and HOMA-IR (*Model 2b*). Additional sensitivity analyses were carried out in participants *with* diabetes, in whom only fasting values of GIP and GLP-1 were available, exploring associations between fasting GIP and GLP-1 and incident first cancer, using Cox-regression models adjusted according to *Model 1* and *Model 2b*. Incident hormone-sensitive cancers were defined as first incident cancer of incident ovarian cancer, endometrial cancer, or incident breast cancer for women, or incident prostate cancer for men. Incident obesity-related cancers were defined as any incident first breast cancer, incident ovarian cancer, incident gastric cancer, incident colorectal cancer, incident endometrial cancer, incident kidney cancer and incident pancreatic cancer. Analyses of incretins association with hormone-sensitive cancers and with obesity-related cancers were carried out in Cox-regressions using *Model 1* and *Model 2a*. In analyses of hormone-sensitive cancers in women, MHT was added on top of *Model 2a*.

### Competing risks analyses

To account for death as a competing risk, Fine-Gray sub-distribution hazard models were used to assess the cumulative incidence of the following endpoints (all incident cancers, hormone- and obesity-related cancers). The multivariable models used were the same as previously mentioned (*Model 1* and *Model 2a*).

### Two-year “lag” analyses

In order to avoid inclusion of cancer cases present but not diagnosed, where findings may be attributed to pre-diagnostic symptoms at the time of the study inclusion, we performed a 2-year “lag” analyses using Fine-Gray sub-distribution hazard models to assess the cumulative incidence of the following endpoints (incident first cancers, hormone-sensitive and obesity-related incident first cancers).

### Analyses of associations between post-OGTT challenge concentrations and incident first cancer

Prior to analyses of associations between post-challenge concentrations and any first cancer, participants with diabetes were excluded (n = 297). Analyses of associations between (1) post-challenge GIP, (2) post-challenge GLP-1, (3) post-challenge glucose, (4) post-challenge insulin and (5) post-challenge glucagon were carried out in *Model 1* and *Model 2a.*

Analyses of specific cancers are described in Supplementary Methods.

All analyses were carried out using SPSS 26.0 (IBM) or STATA 17.0 (StataCorp). All *p*-values were two-tailed. A *p*-value < 0.05 was considered statistically significant. Due to the exploratory nature of this study, nominal *p*-values without adjustment for multiple testing are presented.

## Results

The characteristics of the study population across quintiles of fasting GLP-1 are presented in Table 1. Participants with and without incident cancer are compared in Supplementary Table S1. Participants who suffered from any incident first cancer (n = 485) were younger, more often male, with higher waist circumference and lower post-challenge GIP levels. Further, participants with any incident first cancer were twice as likely to be smokers, and a higher proportion of women who developed incident first cancer were treated with MHT. Comparisons between sexes and between participants with and without diabetes are presented in Supplementary Table S2.

**Table 1:** Characteristics of the study population across quintiles of fasting GLP-1.

	Total	Q1	Q2	Q3	Q4	Q5
Age (years)	72.2 (± 5.6)	72.0 (± 5.8)	72.3 (± 5.7)	72.1 (± 5.7)	72.0 (± 5.6)	72.7 (± 5.3)
Sex (women (n (%))	1720 (59.5)	348 (61.1)	476 (59.4)	238 (59.4)	321 (56.5)	337 (61.1)
BMI (kg/m^2^)	26.9 (4.4)	26.8 (± 4.3)	26.7 (± 4.4)	26.8 (± 4.1)	26.7 (4.3)	27.3 (4.8)
Waist circumference (cm)	92 (12)	92 (± 12)	92 (± 13)	92 (± 12)	92 (± 12)	93 (± 13)
Diabetes status (n (%))	289 (10.0)	36 (6.3)	68 (8.5)	35 (8.7)	46 (8.1)	104 (18.8)
Physical activity (sedentary, n (%))	226 (7.8)	39 (6.8)	60 (7.5)	37 (9.2)	43 (7.6)	47 (8.5)
Incident cancer (n (%))	462 (16.0)	93 (16.3)	141 (17.6)	68 (17.0)	78 (13.7)	82 (14.9)
Glucose^0 min^ (mmol/L)	5.9 (5.4–6.4)	5.9 (5.5–6.4)	5.8 (5.4–6.3)	5.8 (5.3–6.4)	5.9 (5.4–6.4)	5.9 (5.4–6.7)
Glucose^120 min^ (mmol/L)*	6.8 (5.5–8.2)	6.9 (5.6–8.2)	6.8 (5.5–8.1)	6.6 (5.5–8.0)	6.8 (5.5–8.4)	6.8 (5.4–8.5)
GLP-1^0 min^ (pmol/L)	8 (6–10)	4 (3–5)	7 (6–7)	8 (8–8)	9 (9–10)	12 (11–14)
GLP-1^120 min^ (pmol/L)*	16 (12–20)	13 (9–17)	14 (11–18)	16 (13–21)	17 (14–21)	20 (15–26)
GIP^0 min^ (pmol/L)	41 (30–56)	40 (30–53)	40 (29–55)	38 (29–52)	42 (30–58)	47 (34–64)
GIP^120 min^ (pmol/L)*	221 (162–293)	217 (161–294)	220 (160–291)	223 (162–288)	223 (169–300)	218 (153–298)
Insulin^0 min^ (pmol/L)	7.7 (5.4–11.0)	7.4 (5.1–10.8)	7.7 (5.6–10.6)	7.2 (5.2–10.3)	7.7 (5.5–11.1)	8.4 (5.5–12.1)
Insulin^120 min^ (pmol/L)*	41.7 (29.1–60.1)	41.2 (29.5–60.8)	41.6 (29.1–59.3)	41.9 (30.1–60.4)	42.0 (28.7–59.5)	41.4 (28.1–62.9)
Glucagon^0 min^ (pg/mL)	77 (64–92)	76 (64–89)	75 (63–90)	75 (63–90)	77 (64–92)	82 (68–99)
Glucagon^120 min^ (pg/mL)*	70 (59–82)	70 (59–81)	69 (58–81)	68 (58–80)	71 (58–83)	71 (60–85)
Smoking (n (%))	209 (7.2)	36 (6.3)	74 (9.2)	32 (8.0)	33 (5.8)	34 (6.2)
MHT (n (%))^†^	239 (14.6)	48 (13.9)	72 (15.3)	34 (14.5)	42 (7.4)	43 (12.9)

Values are means (± standard deviation) or medians (25–75 interquartile range).

*BMI* body mass index, *MHT* menopausal hormone therapy.

^0 min^Fasting values; ^120 min^oral glucose tolerance test post-challenge values.

*Only available in participants without diabetes.

^**†**^Only available in women.

Spearman rank correlation analyses between all continuous variables explored in this study are presented in Supplementary Table S3.

### Associations of fasting biomarkers with any incident first cancer

Each 1 standard deviation (SD) increment of fasting GLP-1 was associated with decreased risk of incident first cancer during the maximum follow-up period of 12.6 years (median 9.9 years, interquartile range 8.0–11.0 years) in Model 2a (adjusted for age, sex, waist circumference, diabetes status, smoking status and physical activity): HR 0.91 (95% CI 0.83–0.99); P = 0.031 (Table 2). The effect remained stable in competing risk analyses (with death as competing risk): subhazard ratio (SHR) 0.90; 95% CI 0.82–0.99; P = 0.022 (Table 3). Each 1 SD increment of fasting glucose was associated with higher risk of incident first cancer in the *Model 1*, but the association did not remain significant upon further adjustment (Table 2). No associations were seen between fasting insulin or fasting glucagon and incident first cancer.

**Table 2:** Associations between biomarkers of glucose metabolism and any incident first cancer.

	Fasting		Post-challenge	
	GIP			
	451 cases/2324 controls		388 cases/2067 controls	
	HR (95% CI)	p	HR (95% CI)	p
**Model 1**				
GIP	1.09 (0.99–1.20)	0.077	0.97 (0.87–1.07)	0.535
Age	1.00 (0.98–1.01)	0.583	0.99 (0.98–1.01)	0.460
Sex	0.59 (0.49–0.71)	** < 0.001**	0.61 (0.50–0.75)	** < 0.001**
**Model 2a**				
GIP	–	–	–	–
Age	–	–	–	–
Sex	–	–	–	–
Waist circumference	–	–	–	–
Diabetes	–	–	–	–
Smoking	–	–	–	–
Sedentary	–	–	–	–
Moderate exercise	–	–	–	–
Regular exercise	–	–	–	–
Strenuous exercise	–	–	–	–

	GLP-1			
	462 cases/2417 controls		403 cases/2158 controls	
	HR (95% CI)	p	HR (95% CI)	p
**Model 1**				
GLP-1	0.91 (0.84–0.99)	**0.044**	0.93 (0.83–1.03)	0.153
Age	0.99 (0.98–1.01)	0.371	1.00 (0.98–1.01)	0.711
Sex	0.59 (0.49–0.70)	** < 0.001**	0.63 (0.52–0.77)	** < 0.001**
**Model 2a**				
GLP-1	0.91 (0.83–0.99)	0.031	–	–
Age	1.00 (0.98–1.01)	0.616	–	–
Sex	0.62 (0.50–0.76)	** < 0.001**	–	–
Waist circumference	1.00 (1.00–1.01)	0.375	–	–
Diabetes	1.29 (0.97–1.73)	0.084	–	–
Smoking	1.83 (1.37–2.44)	** < 0.001**	–	–
Sedentary	reference	0.793	–	–
Moderate exercise	1.17 (0.79–1.71)	0.432	–	–
Regular exercise	1.13 (0.73–1.75)	0.590	–	–
Strenuous exercise	0.62 (0.08–4.61)	0.643	–	–

	Glucose			
	470 cases/2463 controls		409 cases/2208 controls	
	HR (95% CI)	p	HR (95% CI)	p
**Model 1**				
Glucose	1.12 (1.03–1.23)	0.008	1.08 (0.99–1.19)	0.100
Age	0.99 (0.98–1.01)	0.349	0.99 (0.98–1.01)	0.368
Sex	0.62 (0.51–0.74)	** < 0.001**	0.61 (0.50–0.74)	** < 0.001**
**Model 2a**				
Glucose	1.11 (1.00–1.25)	0.059	–	–
Age	1.00 (0.98–1.01)	0.602	–	–
Sex	0.63 (0.51–0.77)	** < 0.001**	–	–
Waist circumference	1.00 (0.99–1.01)	0.605	–	–
Diabetes	1.05 (0.74–1.49)	0.798	–	–
Smoking	1.83 (1.37–2.43)	** < 0.001**	–	–
Sedentary	Reference	0.791	–	–
Moderate exercise	1.18 (0.80–1.73)	0.411	–	–
Regular exercise	1.15 (0.75–1.79)	0.515	–	–
Strenuous exercise	0.64 (0.09–4.71)	0.658	–	–

	Insulin			
	451 cases/2328 controls		388 cases/2067 controls	
	HR (95% CI)	p	HR (95% CI)	p
**Model 1**				
Insulin	1.06 (0.96–1.16)	0.230	1.05 (0.96–1.16)	0.298
Age	1.00 (0.98–1.01)	0.615	0.99 (0.97–1.01)	0.346
Sex	0.59 (0.49–0.71)	** < 0.001**	0.60 (0.49–0.74)	** < 0.001**
**Model 2a**				
Insulin	–	–	–	–
Age	–	–	–	–
Sex	–	–	–	–
Waist circumference	–	–	–	–
Diabetes	–	–	–	–
Smoking	–	–	–	–
Sedentary	–	–	–	–
Moderate exercise	–	–	–	–
Regular exercise	–	–	–	–
Strenuous exercise	–	–	–	–

	Glucagon			
	467 cases/2450 controls		407 cases/2192 controls	
	HR (95% CI)	p	HR (95% CI)	p
**Model 1**				
Glucagon	1.08 (0.98–1.19)	0.121	1.07 (0.96–1.18)	0.212
Age	0.99 (0.98–1.01)	0.358	0.99 (0.98–1.01)	0.528
Sex	0.61 (0.51–0.74)	** < 0.001**	0.61 (0.50–0.74)	** < 0.001**
**Model 2a**				
Glucagon	–	–	–	–
Age	–	–	–	–
Sex	–	–	–	–
Waist circumference	–	–	–	–
Diabetes	–	–	–	–
Smoking	–	–	–	–
Sedentary	–	–	–	–
Moderate exercise	–	–	–	–
Regular exercise	–	–	–	–
Strenuous exercise	–	–	–	–

Values are hazard ratios (HR) with 95% confidence intervals (95%CI) per 1SD increment in biomarkers. Associations between post-challenge biomarkers and are carried out in participants without diabetes only. Model 1 is adjusted for age and sex. Model 2a is adjusted for age, sex, waist circumference, smoking status, diabetes status and physical activity. Analyses of post-challenge biomarkers were carried out in participants without diabetes, and adjusted for age, sex, waist circumference, smoking and physical activity).

Significant values are in bold.

**Table 3:** Associations between fasting GIP and GLP-1, and hormone-sensitive and obesity-related incident first cancers.

Hormone-sensitive cancers				Obesity-related cancers	
Fasting GIP					
Men		Women			
96 cases/1038 controls		72 cases/1556 controls		173 cases/2600 controls	
HR (95%CI)	p	HR (95%CI)	p	HR (95%CI)	p
0.99 (0.80–1.22)	0.940	1.17 (0.92–1.48)	0.208	1.09 (0.93–1.27)	0.287

Fasting GLP-1					
98 cases/1070 controls		71 cases/1628 controls		172 cases/2705 controls	
0.89 (0.73–1.10)	0.279	0.85 (0.70–1.05)	0.125	0.94 (0.81–1.08)	0.393

Post-challenge GIP					
80 cases/858 controls		63 cases/1336 controls		142 cases/2200 controls	
0.90 (0.72–1.13)	0.357	1.20 (0.91–1.59)	0.195	1.06 (0.89–1.27)	0.514

Post-challenge GLP-1					
79 cases/884 controls		65 cases/1395 controls		150 cases/2275 controls	
0.82 (0.63–1.06)	0.123	1.10 (0.85–1.42)	0.464	1.02 (0.85–1.21)	0.870

Values are hazard ratios (HR) with 95% confidence intervals (95%CI) adjusted according to Model 2a for fasting values (age, sex, diabetes, waist circumference, smoking status and physical activity), and according to Model 2b for post-challenge values (age, sex, waist circumference, smoking status, HOMA-IR and physical activity). In analyses of hormone-sensitive cancers in women, hormone replacement therapy was added to the adjustment model. Incident hormone-sensitive cancers are defined as first incident cancer of any of incident ovarian cancer, endometrial cancer, incident breast cancer for women, and incident prostate cancer for men. Incident obesity-related cancers are defined as any incident first breast cancer, incident ovarian cancer, incident gastric cancer, incident colorectal cancer, incident endometrial cancer, incident kidney cancer and incident pancreatic cancer.

### Sensitivity analyses

Sensitivity analyses carried out in participants without diabetes showed no significant associations between fasting GIP and incident first cancer (p = 0.419) in *Model 2b*). Fasting GLP-1 was associated with lower risk of incident first cancer in participants without diabetes (HR 0.91, 95% CI 0.83–0.99; p = 0.048) when adjusted for age, sex, waist circumference, and smoking status but the association was attenuated when physical activity was entered upon *Model 2b* (p = 0.052; Supplementary Table S4). None of fasting glucose, insulin and glucagon showed significant associations with incident first cancer in the sensitivity analyses (Supplementary Table S4).

### Associations of post-challenge biomarkers with any incident first cancer

Prior to analyses of associations between post-challenge biomarkers and incident first cancer, participants with diabetes were excluded (n = 297). No associations were seen for any of the post-challenge incretins and incident first cancer (Table 2) in the adjusted models. For participants without diabetes, there were also no associations between post-challenge glucose, post-challenge insulin and post-challenge glucagon (Supplementary Table S4) with incident first cancer.

### Incretins’ associations with hormone-sensitive and obesity-related incident primary cancers

No sex-specific associations were seen between any of fasting GIP, fasting GLP-1, post-OGTT challenge GIP and post-OGTT challenge GLP-1 and hormone-sensitive cancers. No associations with incident obesity-related cancers were seen for either fasting GIP or GLP-1 (Table 3) when analyzed using the complete follow-up duration. However, in analyses of obesity-related cancers, fasting GLP-1 levels were associated with lower risk (HR 0.80, 95%CI 0.65–0.99; p = 0.042) of obesity-related cancers for each 1 SD increment for the first 4 years, but after that no such association was seen (HR 1.04, 95%CI 0.86–1.25; p = 0.690). The number of cases/controls for analyses of fasting GIP and GLP-1 and specific cancers is presented in Supplementary Table S5. There were no significant associations between fasting GIP and GLP-1, and the most common cancers, i.e., colorectal, breast, and prostate cancer in univariable analyses (Supplementary Table S6), therefore, no further analyses were conducted.

### Competing risk analyses

In the competing risk analyses, the effect estimates remained essentially the same as in the original Cox models, indicating a stable effect size when considering death as competing risk to any incident cancer (Table 4).

**Table 4:** Associations between fasting and post-challenge incretins and any incident first cancer in competing risk regression.

	Fasting GIP		Post-challenge GIP	
	SHR (95%CI)	p	SHR (95%CI)	p
Fasting GIP	1.05 (0.95–1.15)	0.344	0.95 (0.85–1.05)	0.288
Age	0.99 (0.97–1.00)	0.124	0.99 (0.97–1.00)	0.109
Sex	0.66 (0.53–0.81)	** < 0.001**	0.68 (0.54–0.85)	**0.001**
Waist circumference	1.00 (1.00–1.01)	0.310	1.00 (0.99–1.01)	0.522
Diabetes	1.17 (0.88–1.57)	0.279	–	–
Smoking	1.76 (1.31–2.37)	** < 0.001**	1.87 (1.37–2.55)	** < 0.001**
Sedentary	Reference		Reference	
Moderate exercise	1.27 (0.87–1.86)	0.218	1.31 (0.85–2.00)	0.220
Regular exercise	1.32 (0.86–2.02)	0.207	1.26 (0.78–2.04)	0.344
Strenuous exercise	0.74 (0.12–4.77)	0.756	0.77 (0.12–4.93)	0.782

	Fasting GLP-1		Post-challenge GLP-1	
	SHR (95%CI)	p	SHR (95%CI)	p
Fasting GLP-1	0.90 (0.82–0.99)	**0.022**	0.93 (0.85–1.05)	0.159
Age	0.98 (0.97–1.00)	**0.041**	0.99 (0.97–1.00)	0.189
Sex	0.64 (0.52–0.79)	** < 0.001**	0.68 (0.54–0.85)	** < 0.001**
Waist circumference	1.00 (1.00–1.01)	0.433	1.00 (0.99–1.01)	0.806
Diabetes	1.24 (0.93–1.66)	0.146	–	–
Smoking	1.75 (1.31–2.34)	** < 0.001**	1.87 (1.32–2.41)	** < 0.001**
Sedentary	Reference		Reference	
Moderate exercise	1.28 (0.88–1.89)	0.191	1.36 (0.89–2.08)	0.157
Regular exercise	1.27 (0.83–1.96)	0.269	1.25 (0.77–2.00)	0.365
Strenuous exercise	0.71 (0.11–4.68)	0.718	0.79 (0.12–5.17)	0.806

Values are sub-hazard ratios (SHR) with 95% confidence intervals (95 CI%) per 1SD increment in biomarkers. Associations between post-challenge biomarkers and are carried out in participants without diabetes only. Analyses of fasting biomarkers are adjusted for age, sex, waist circumference, smoking, diabetes status and physical activity. Analyses of post-challenge biomarkers are carried out in participants without diabetes and thus adjusted for age, sex, waist circumference, smoking, HOMA-IR and physical activity.

Significant values are in bold.

### Two-year “lag” analyses

For the 2-year lag analysis, there were 2891 participants with available follow-up time and among them, 375 cases of incident first cancer. The effect sizes for all analyses remained essentially the same but the confidence intervals got wider due to the lower number of cases (Supplementary Tables S7, S8).

### Incretin’s associations with any incident first cancer in participants with diabetes

In participants with diabetes, in whom only fasting concentrations of GIP and GLP-1 were available, no unadjusted associations between fasting incretin GIP (56 first incident cancers/228 controls; HR 1.09; 95% CI 0.84–1.42; p = 0.504) and fasting GLP-1 (54 first incident cancers/225 controls; HR 0.87; 95% CI 0.68–1.10; p = 0.248) and incident first cancer were observed but the numbers were small.

Further, as several cancers might present with unexpected weight loss, we carried out additional analyses with participants categorized into 1) underweight (BMI ≤ 18.5), normal BMI (BMI 18.5–24.9), overweight (25–29.9) and obese (≥ 30) and found no increased risk of first incident cancer in underweight participants (HR 0.96; 95%CI 0.71–1.30; p = 0.777).

## Discussion

In this explorative, population-based study of elderly participants, originally free from prevalent cancers and followed for a median of 9.9 years, we found no increased risk of incident first cancer associated with either fasting or post-OGTT challenge GIP and GLP-1. In addition, higher endogenous fasting GLP-1 levels were found to be associated with lower risk of any incident first cancer, whereas both fasting glucose and GIP-levels were marginally significantly associated with increased risk of incident cancer risk when adjusted for age and sex. These associations were attenuated upon further adjustment.

Further, fasting GIP was neither associated with obesity-related, nor hormone-sensitive cancers. In contrast, fasting GLP-1 was associated with a lower risk of obesity-related cancers during the first 4 years of follow-up and a lower risk of hormone-sensitive cancers during the entire follow-up. Those findings might be attributed to the possible role of GLP-1 as an inhibitor of cancer cell proliferation^13^, whereas no effect on tumor initiation has been reported. In addition, several cancer types defined as obesity-related in our work are gastrointestinal cancers, whose presence in the gastrointestinal tract might alter GLP-1 secretion by disturbing enteroendocrine cell stimulation^21^.

Competing risk (death as competing risk) analyses indicated that the effect was stable. To our knowledge, this is the first study to explore the associations of endogenous incretin levels and incident first cancer.

There might be several plausible explanations for the observed associations between higher endogenous GLP-1 levels and lower risk of incident cancer. The mechanisms are probably multifactorial and complex. In our study, we found no significant association between GLP-1 and reduced risk of breast cancer, but this might be attributable to the low event rate. We did not analyze associations with prevalent cancer in our study and excluded participants with a cancer diagnosis at study entrance since we had no information on disease status or treatment.

Previous findings regarding possible pro-oncogenic characteristics of GLP-1 RA have been inconsistent. Several studies have shown that incretin-based drugs are associated with increased risk of multiple cancer types^8,22–24^. A recent real-world study using FAERS data from the first quarter of 2004 to the second quarter of 2020 reported significant signals between GLP-1RA and certain tumors, including medullary thyroid cancer, papillary thyroid cancer, malignant pancreatic neoplasms, islet cell neoplasms and APUDoma NEC. They suggested the combination of GLP-1 RA and DPP4i might have caused the increased reporting rate in tumors^6^. On the contrary, Ruette et al*.* did not find any increased risk of lung cancer for users of DPP4i and GLP-1 RA, compared to users of second-/third line drugs^25^. Further, it has been shown that GLP-1 RA can inhibit breast cancer cells growth^13^, and two recent meta-analyses concluded that there was no evidence of GLP-1 RA usage and increased risk of biliary tract cancer^26^, or malignant neoplasia^27^. Our observed neutral or cancer-protective associations between endogenous incretin levels and incident first cancer should be viewed in the light of the fact that associations between endogenous levels of incretins and incident first cancer were explored here, whereas incretin-based drug usage results in supraphysiological incretin levels. Overweight/obesity is a condition more prevalent in participants with diabetes, and an established risk factor for cancer^28^. However, in this study population, there was no significant difference in BMI between participants with and without incident cancer. The BMI presented here is, however, measured at one specific point in time (baseline), and we had no follow-up data on BMI, which changes over the years. Participants with, and without diabetes, however, differed in almost all aspects except for age, smoking, MHT-usage, and cancer incidence. Supraphysiological GLP-1 levels achieved by GLP-1 agonist treatment have been shown to exert protective effects on β-cells^29–31^. However, our study does not offer insight into whether GLP-1 is protective with regard to tumor initiation, or inhibited promotion of existing tumors. Nevertheless, given that it takes 10–30 years from tumor initiation to detectable cancer^32^, and that the current study population had a mean age of > 70 years, GLP-1 might, if anything, have inhibiting effects on existing tumor promotion.

We carried out our analyses of fasting incretins in all participants, irrespective of diabetes and additionally, separately in participants with and without diabetes, as individuals with diabetes are at significantly higher risk of developing many forms of cancer^33^. The sensitivity analysis in participants without diabetes showed similar results, with fasting GLP-1 levels borderline associated with lower risk of any incident cancer. Due to the known sex-specific differences in cancer incidence^34–36^ with approximately 20% higher cancer incidence in men^36^, we carried out additional sex-specific analyses for associations between incretins and risk of any incident cancer, finding a borderline association between higher fasting GLP-1 and lower cancer risk only in men.

### Strengths and limitations

One strength of this study is the relatively large study population, followed for a median of 9.9 years. Further, we used validated and robust national registries of high quality and data completeness (96%)^37–39^. We carried out an OGTT in participants free from known diabetes at baseline, allowing us to be certain about participants’ diabetes status. In this context, this is of great importance, since the GLP-1 response to OGTT may be reduced in established type 2 diabetes and obesity^40^, while GIP secretion is near normal in diabetes, but its effect on insulin secretion is impaired^41^.

A limitation of this study is the low number of events in analyses of specific cancers, in particular in analyses regarding risk of pancreatic cancer, which would have been of particular interest to explore. However, with an event frequency of 0.4%, no meaningful analyses could be carried out. According to the Swedish Cancer Registry^20^, there is a delay of approximately 5% in general reporting coverage, making it comparable to other high quality registers in northern Europe. However, a comparison between the Cancer Registry data and the Cause of Death Registry shows that underreporting is somewhat dependent on the cancer site, where pancreatic and lung cancers tend to be missed more often than breast cancer^20^. Another weakness was the many missing variables on self-reported alcohol consumption. Therefore, we were not able to adjust for alcohol consumption, which is a risk factor for cancer. Multiple imputation to account for missing data on alcohol consumption was deemed inappropriate since data were assumed to be missing not at random. Also, survivorship bias must be considered in this setting, resulting in participants that did not survive long enough to attend the re-examination study of the MDC-CC being excluded. In addition, physical activity was self-reported, and although this question had high response rate, individuals may overestimate or underestimate their true rates of activity^42^.

Further, being an observational study, we cannot draw any conclusions about causality. Our participants were of mostly white European descent and within a narrow older age range, therefore the conclusions may not be generalizable to other populations or age groups.

## Conclusions

In this prospective, community-based study, no associations with higher risk of incident cancer were observed either for fasting or for post-challenge endogenous GIP and GLP-1. In addition, elevated levels of endogenous fasting GLP-1 were associated with lower risk of any incident first cancer within the 12.6 years of follow up. Further studies exploring incretins’ associations with cancer are warranted.

## Supplementary Information

Supplementary Information.

## Data Availability

The data that support the findings of this study are available upon request from Steering Committee of Malmö Diet and Cancer study by contacting data manager Anders Dahlin (anders.dahlin@med.lu.se), but restrictions apply to the availability of these data, which were used under license for the current study, and so are not publicly available due to ethical and legal restrictions related to the Swedish Biobanks in Medical Care Act (2002:297) and the Personal Data Act (1998:204).

## Abbreviations

BMI: Body mass index
DPP4i: Dipeptidyl peptidase-4 inhibitors
FAERS: FDA adverse event reporting system
FPG: Fasting plasma glucose
GIP: Glucose-dependent insulinotropic peptide
GLP-1: Glucagon-like peptide 1
GLP-1 RA: Glucagon-like peptide 1 receptor agonists
HOMA-IR: Homeostatic Model Assessment for Insulin Resistance
MDC-CC: The Malmö Diet and Cancer Cardiovascular Cohort
MHT: Menopausal hormone therapy
NEC: Neuroendocrine cancer
OGTT: Oral glucose tolerance test
