# Supplementary material for: Endogenous incretin levels and risk of first incident cancer: a prospective cohort study
Source: Sci Rep. 2023 Jan 7;13:382. doi: 10.1038/s41598-023-27509-3 (PMC9825393; doi:10.1038/s41598-023-27509-3)
Supplement: Supplementary file 1 — Supplementary Information. [file 41598_2023_27509_MOESM1_ESM.docx]

**Endogenous incretin levels and risk of first incident cancer - a prospective cohort study**

**SUPPLEMENTARY MATERIAL**

Amra Jujić, PhD^1,2,3*^, Christopher Godina, MMedSci^4^, Mattias Belting, PhD^4,5^, Olle Melander, PhD^3^, Jens Juul Holst, PhD^6,7^, Emma Ahlqvist, PhD^3^, Maria F. Gomez, PhD^3^, Peter M. Nilsson, PhD^1^, Helena Jernström, PhD§^4^ and Martin Magnusson, PhD§^1,2,8,9^

^1^ Department of Clinical Sciences Malmö, Lund University, Sweden

^2^ Department of Cardiology, Skåne University Hospital, Sweden

^3^ Lund University Diabetes Centre, Department of Clinical Sciences, Lund University, Malmö, Sweden

^4^ Department of Clinical Sciences Lund, Oncology, Lund University and Skåne University Hospital, Lund, Sweden

^5^ Department of Immunology, Genetics, and Pathology, Science for Life Laboratory, Uppsala University, Uppsala, Sweden

^6^ Department of Biomedical Sciences and NNF Center for Basal Metabolic Research, The Panum Institute, Copenhagen, Denmark

^7^ NNF Center for Basal Metabolic Research, University of Copenhagen, Denmark

^8^ Wallenberg Center for Molecular Medicine, Lund University, Malmö, Sweden.

^9^ Hypertension in Africa Research Team (HART), North West University Potchefstroom, South Africa

§Shared senior authorship

***Corresponding author:** Amra Jujić; Phone: +46739289857; Lund University, Clinical Research Centre, Box 50332, 202 13 Malmö, Sweden. e-mail: [amra.jujic@med.lu.se](mailto:amra.jujic@med.lu.se)

Contents

[Supplementary Methods 3](#_Toc122521626)

[Supplementary Table S1. Characteristics of the study population with and without incident first cancer 4](#_Toc122521627)

[Supplementary Table S2. Characteristics of the study population, comparison between sex and between subjects with and without diabetes 5](#_Toc122521628)

[Supplementary Table S3. Spearman rank correlations between markers of metabolic health 6](#_Toc122521629)

[Supplementary Table S4. Sensitivity analyses excluding participants with diabetes 8](#_Toc122521630)

[Supplementary Table S5. Number of cases/controls for analyses of individual cancers 9](#_Toc122521631)

[Supplementary Table S6. Unadjusted associations between fasting GIP and GLP-1 and colorectal, breast, and prostate cancers 10](#_Toc122521632)

[Supplementary Table S7. (2-year lag analysis) Adjusted associations between fasting GIP and GLP-1 and any incident primary cancer in competing risk regression with 2-year lag 11](#_Toc122521633)

[Supplementary Table S8. (2-year lag analysis) Adjusted associations between post-challenge GIP and GLP-1 and any incident primary cancer in competing risk regression with 2-year lag 12](#_Toc122521634)

# Supplementary Methods

*Analyses of specific cancers*

Multiple univariate Cox regressions were carried out for three different cancer diagnoses (≥2% event frequency), i.e. colorectal, breast, and prostate cancer. To explore possible hormone-modifying effects of incretins, hormone-sensitive incident cancers (incident ovarian cancer (n=6); incident corpus uteri cancer (n=13), incident breast cancer (n=56) in women were pooled prior to analyses. In men, incident prostate cancer (n=98) was defined as hormone-sensitive cancer. Obesity-related incident cancers (postmenopausal breast cancer (n=56), ovarian cancer (n=6), gastric cancer (n=11), colorectal cancer (n=71), endometrial cancer (n=14), kidney cancer (n=7), and pancreatic cancer (n=12)) were pooled prior to analyses.

# Supplementary Table S1. Characteristics of the study population with and without incident first cancer

|  | **Subjects without incident cancer** | **Subjects with incident cancer** | **p** |
| --- | --- | --- | --- |
| **Age (years)** | 72.3 (±5.7) | 71.5 (±5.3) | **0.004** |
| **Sex (women (n (%))** | 1564 (61.0) | 233 (48.0) | **1.0x10^-7^** |
| **BMI (kg/m^2^)** | 26.8 (±4.3) | 27.1 (±4.5) | 0.154 |
| **Waist circumference (cm)** | 92.1 (±12.4) | 94.0 (±12.2) | **0.002** |
| **Diabetes status (n (%))** | 257 (10) | 59 (12.2) | 0.156 |
| **Glucose^0min^ (mmol/L)** | 5.8 (5.4–6.4) | 6.0 (5.5–6.6) | **0.002** |
| **Glucose^120min^ (mmol/L)** | 6.8 (5.5–8.2) | 6.7 (5.6–8.8) | 0.292 |
| **GLP-1^0min^ (pmol/L)** | 8 (6–10) | 7.5 (6–10) | 0.105 |
| **GLP-1^120min^ (pmol/L)** | 16 (12–20) | 15 (11–20) | **0.005** |
| **GIP^0min^ (pmol/L)** | 40.8 (30–56) | 43.3 (31–59) | 0.078 |
| **GIP^120min^ (pmol/L)** | 223 (163–292) | 211 (154–293) | 0.159 |
| **Insulin^0min^ (pmol/L)** | 7.6 (5.4–10.9) | 8.2 (5.8–11.4) | 0.060 |
| **Insulin^120min^ (pmol/L)** | 38.8 (25.4–62.7) | 40.9 (26.8–63.2) | 0.397 |
| **Glucagon^0min^ (pg/ml)** | 76.0 (64–91) | 79.9 (65.4–94.1) | **0.003** |
| **Glucagon^120min^ (pg/ml)** | 69.0 (58.0–82.0) | 71.4 (60.0–83.3) | 0.061 |
| **Smoking (n (%))** | 162 (6.3) | 54 (11.1) | **1.7x10^-4^** |
| **HRT (n (%) women only)** | 627 (40.1) | 111 (47.6) | **0.027** |
|  |  |  |  |

# Supplementary Table S2. Characteristics of the study population, comparison between sex and between subjects with and without diabetes

|  | **Total** | **Men** | **Women** | **p** | **Participants without**  **diabetes** | **Participants with**  **diabetes** | **p** |
| --- | --- | --- | --- | --- | --- | --- | --- |
|  | **n=2948** | **n=1194** | **n=1754** |  | **n=2651** | **n=297** |  |
| **Age (years)** | 72.2 (±5.6) | 71.9 (±5.7) | 72.4 (±5.6) | **0.016** | 72.1 (±5.6) | 72.7 (±5.4) | 0.117 |
| **Sex (women (n (%))** | 1754 (59.5) | – | – | – | 1643 (60.1) | 154 (48.7) | **9.9x10^-5^** |
| **BMI (kg/m^2^)** | 26.9 (±4.4) | 27.1 (±3.9) | 26.7 (±4.7) | **1.1x10^-4^** | 26.6 (±4.2) | 29.2 (±5.2) | **1.1x10^-19^** |
| **Waist circumference (cm)** | 92.3 (±12.5) | 98.8 (±10.2) | 87.8 (±11.9) | **2.6x10^-130^** | 91.5 (±12.1) | 100.1 (±12.8) | **1.5x10^-26^** |
| **Diabetes status (n (%))** | 297 (10.1) | 162 (12.9) | 154 (8.6) | **9.9x10^-5^** | – | – | – |
| **Incident cancer (n (%))** | 470 (15.9) | 252 (20.1) | 233 (13.0) | **1.0x10^-7^** | 426 (15.6) | 59 (18.7) | 0.156 |
| **Glucose^0min^ (mmol/L)** | 5.9 (5.4-6.4) | 6.0 (5.5-6.6) | 5.8 (5.3-6.3) | **1.0x10^-13^** | 5.8 (5.4-6.2) | 8.1 (6.9-9.7) | **9.5x10^-111^** |
| **Glucose^120min^ (mmol/L)*** | – | – | – | – | 6.8 (5.5-8.2) | – | – |
| **GLP-1^0min^ (pmol/L)** | 8 (6-10) | 8 (6-10) | 8 (6-10) | 0.519 | 8 (6.10) | 9 (7-12) | **2.7x10^-11^** |
| **GLP-1^120min^ (pmol/L)*** | – | – | – | – | 16 (12-20) | – | – |
| **GIP^0min^ (pmol/L)** | 40.8 (30.3-56.6) | 41.1 (30-56) | 41 (30-57) | 0.911 | 40 (30-54) | 53 (38-77) | **1.1x10^-20^** |
| **GIP^120min^ (pmol/L)*** | – | – | – | – | 220 (162-293) | – | – |
| **Insulin^0min^ (pmol/L)** | 53.5 (37.5-76.4) | 57.0 (39.6-79.1) | 51.4 (36.8-74.3) | **9.0x10^-6^** | 52.1 (36.8-73.6) | 73.6 (44.4-100.0) | **2.4x10^-15^** |
| **Insulin^120min^ (pmol/L)*** | – | – | – | – | 277.1 (177.8-435.4) | – | – |
| **Glucagon^0min^ (pg/ml)** | 77 (64-92) | 84 (71-99) | 73 (61-86) | **4.5x10^-46^** | 75.3 (63.4-90.0) | 90.6 (76.0-108.0) | **1.2x10^-26^** |
| **Glucagon^120min^ (pg/ml)*** | – | – | – | – | 69.8 (58.3-82.0) | – | – |
| **Smoking (n (%))** | 289 (9.8) | 93 (7.4) | 123 (6.8) | 0.427 | 200 (7.3) | 16 (5.1) | 0.169 |
| **HRT (n (%))†** | – | – | 738 (41.4) | – | 156 (42.0) | 14 (31.8) | 0.146 |

Values are means (± standard deviation) or medians (25-75 interquartile range). ^0min^ – fasting values; ^120min^ – oral glucose tolerance test post-challenge values; BMI – body mass index; HRT – hormone replacement therapy. *Only available in participants without diabetes; **†** only available in women

# Supplementary Table S3. Spearman rank correlations between markers of metabolic health

|  | | GIP^f^ | GIP^120min^ | GLP-1^f^ | GLP-1^120min^ | Glucagon^f^ | Glucagon^120min^ | Insulin^f^ | Insulin^120min^ | Glucose^f^ | Glucose^120min^ | Age | Waist  circumference |
| --- | --- | --- | --- | --- | --- | --- | --- | --- | --- | --- | --- | --- | --- |
| GIP^f^ | rho |  | 0.371 | 0.099 | -0.038 | 0.233 | 0.130 | 0.205 | 0.082 | 0.124 | 0.063 | 0.099 | 0.105 |
|  | p |  | **2.3x10^-79^** | **1.3x10^-7^** | 0.059 | **2.9x10^-36^** | **4.4x10^-11^** | **2.4x10^-28^** | **6.0x10^-5^** | **2.6x10^-11^** | **0.002** | **1.2x10^-7^** | **1.7x10^-8^** |
| GIP^120min^ | rho | 0.371 |  | 0.021 | 0.204 | 0.039 | 0.108 | -0.006 | 0.160 | -0.019 | 0.057 | 0.193 | -0.147 |
|  | p | **2.3x10^-79^** |  | 0.304 | **1.0x10^-24^** | **0.048** | **5.3x10^-8^** | 0.779 | **6.6x10^-16^** | 0.338 | **0.004** | **1.0x10^-22^** | **1.2x10^-13^** |
| GLP-1^f^ | rho | 0.099 | 0.021 |  | 0.389 | 0.115 | 0.032 | 0.060 | -0.014 | 0.028 | -0.012 | 0.011 | 0.034 |
|  | p | **1.3x10^-7^** | 0.304 |  | **4.8x10^-96^** | **3.4x10^-10^** | 0.105 | **0.002** | 0.499 | 0.123 | 0.528 | 0.536 | 0.062 |
| GLP-1^120min^ | rho | -0.038 | 0.204 | 0.389 |  | -0.113 | 0.119 | -0.151 | -0.047 | -0.140 | -0.120 | 0.170 | -0.260 |
|  | p | 0.059 | **1.0x10^-24^** | **4.8x10^-96^** |  | **6.6x10^-9^** | **1.1x10^-9^** | **2.7x10^-14^** | **0.018** | **5.5x10^-13^** | **7.8x10^-10^** | **1.6x10^-18^** | **6.6x10^-42^** |
| Glucagon^f^ | rho | 0.233 | 0.039 | 0.115 | -0.113 |  | 0.704 | 0.346 | 0.221 | 0.173 | 0.155 | 0.044 | 0.322 |
|  | p | **2.9x10^-36^** | **0.048** | **3.4x10^-10^** | **6.6x10^-9^** |  | **<0.001** | **6.4x10^-81^** | **3.1x10^-29^** | **1.2x10^-21^** | **5.8x10^-16^** | **0.016** | **3.6x10^-73^** |
| Glucagon^120min^ | rho | 0.130 | 0.108 | 0.032 | 0.119 | 0.704 |  | 0.115 | 0.046 | 0.065 | -0.017 | 0.082 | 0.114 |
|  | p | **4.4x10^-11^** | **5.3x10^-8^** | 0.105 | **1.1x10^-9^** | **<0.001** |  | **6.2x10^-9^** | **0.021** | **0.001** | 0.375 | **2.3x10^-5^** | **3.0x10^-9^** |
| Insulin^f^ | rho | 0.205 | -0.006 | 0.060 | -0.151 | 0.346 | 0.115 |  | 0.651 | 0.340 | 0.275 | 0.012 | 0.503 |
|  | p | **2.4x10^-28^** | 0.779 | **0.002** | **2.7x10^-14^** | **6.4x10^-81^** | **6.2x10^-9^** |  | **1.1x10^-290^** | **2.7x10^-78^** | **1.7x10^-45^** | 0.531 | **9.3x10^-183^** |
| Insulin^120min^ | rho | 0.082 | 0.160 | -0.014 | -0.047 | 0.221 | 0.046 | 0.651 |  | 0.285 | 0.554 | 0.122 | 0.313 |
|  | p | **6.0x10^-5^** | **6.6x10^-16^** | 0.499 | **0.018** | **3.1x10^-29^** | **0.021** | **1.1x10^-290^** |  | **2.7x10^-48^** | **6.7x10^-203^** | **7.2x10^-10^** | **1.7x10^-58^** |
| Glucose^f^ | rho | 0.124 | -0.019 | 0.028 | -0.140 | 0.173 | 0.065 | 0.340 | 0.285 |  | 0.412 | 0.044 | 0.286 |
|  | p | **2.6x10^-11^** | 0.338 | 0.123 | **5.5x10^-13^** | **1.2x10^-21^** | **0.001** | **2.7x10^-78^** | **2.7x10^-48^** |  | **9.0x10^-111^** | **0.016** | **5.5x10^-58^** |
| Glucose^120min^ | rho | 0.063 | 0.057 | -0.012 | -0.120 | 0.155 | -0.017 | 0.275 | 0.554 | 0.412 |  | 0.180 | 0.178 |
|  | p | **0.002** | **0.004** | 0.528 | **7.8x10^-10^** | **5.8x10^-16^** | 0.375 | **1.7x10^-45^** | **6.7x10^-203^** | **9.0x10^-111^** |  | **3.9x10^-21^** | **1.5x10^-20^** |
| Age | rho | 0.099 | 0.193 | 0.011 | 0.170 | 0.044 | 0.082 | 0.012 | 0.122 | 0.044 | 0.180 |  | -0.059 |
|  | p | **1.2x10^-7^** | **1.0x10^-22^** | 0.536 | **1.6x10^-18^** | **0.016** | **2.3x10^-5^** | 0.531 | **7.2x10^-10^** | **0.016** | **3.9x10^-21^** |  | **0.001** |
| Waist  circumference | rho | 0.105 | -0.147 | 0.034 | -0.260 | 0.322 | 0.114 | 0.503 | 0.313 | 0.286 | 0.178 | -0.059 |  |
|  | p | **1.7x10^-8^** | **1.2x10^-13^** | 0.062 | **6.6x10^-42^** | **3.6x10^-73^** | **3.0x10^-9^** | **9.3x10^-183^** | **1.7x10^-58^** | **5.5x10^-58^** | **1.5x10^-20^** | **0.001** |  |

f = fasting concentrations

120min = post-challenge concentrations

# Supplementary Table S4. Sensitivity analyses excluding participants with diabetes

|  | **HR (CI95%)** | **p** |
| --- | --- | --- |
| **FASTING GIP** | 1.04 (0.94–1.16) | 0.419 |
| **POST-CHALLENGE GIP** | 0.98 (0.88-1.09) | 0.655 |
| **FASTING GLP-1** | 0.91 (0.83–1.00) | 0.052 |
| **POST-CHALLENGE GLP-1** | 0.94 (0.84.-1.05) | 0.260 |
| **FASTING GLUCOSE** | 1.16 (0.98–1.37) | 0.079 |
| **POST-CHALLENGE GLUCOSE** | 1.09 (0.98-1.21) | 0.104 |
| **FASTING INSULIN** | 0.65 (0.41–1.05) | 0.079 |
| **POST-CHALLENGE INSULIN** | 1.07 (0.93-1.22) | 0.345 |
| **FASTING GLUCAGON** | 1.07 (0.96–1.19) | 0.259 |
| **POST-CHALLENGE GLUCAGON** | 1.06 (0.96-1.17) | 0.283 |

Values are hazard ratios for Model 2b (adjusted for age, sex, waist circumference, smoking status, HOMA-IR and physical activity.

# Supplementary Table S5. Number of cases/controls for analyses of individual cancers

|  | Total | Cases | % | Censored |
| --- | --- | --- | --- | --- |
| Incident gastric cancer | 2846 | 11 | 0.4 | 2835 |
| **Incident colorectal cancer** | 2890 | 70 | 2.3 | 2820 |
| Incident pancreatic cancer | 2855 | 12 | 0.4 | 2843 |
| Incident lung cancer | 2885 | 35 | 1.1 | 2850 |
| **Incident breast cancer** | 1698 | 53 | 2.9 | 1645 |
| Incident cervix cancer | 1350 | 2 | 0.1 | 1348 |
| Incident endometrial cancer | 1697 | 12 | 0.7 | 1685 |
| Incident ovarian cancer | 1699 | 6 | 0.3 | 1693 |
| **Incident prostate cancer** | 1172 | 98 | 7.8 | 1074 |
| Incident kidney cancer | 2821 | 7 | 0.2 | 2814 |
| Incident urinary cancer | 2880 | 31 | 1.0 | 2849 |
| Incident malignant melanoma | 2879 | 36 | 1.2 | 2843 |
| Incident cancer in the nervous system | 2884 | 7 | 0.2 | 2877 |
| Incident malignant lymphoma | 2860 | 6 | 0.2 | 2854 |

**Bold font type** represents cancers that were taken forward to analyses (≥2% cases).

# Supplementary Table S6. Unadjusted associations between fasting GIP and GLP-1 and colorectal, breast, and prostate cancers

| **FASTING GIP** | | |
| --- | --- | --- |
|  | **HR (CI95%)** | **p** |
| **Incident colorectal cancer** | 1.13 (0.89–1.44) | 0.303 |
| **Incident breast cancer** | 1.07 (0.81–1.41) | 0.625 |
| **Incident prostate cancer** | 0.95 (0.78–1.17) | 0.880 |
|  |  |  |
| **FASTING GLP-1** | | |
|  |  |  |
| **Incident colorectal cancer** | 0.98 (0.80–1.21) | 0.581 |
| **Incident breast cancer** | 0.80 (0.64–1.01) | 0.064 |
| **Incident prostate cancer** | 0.94 (0.78–1.14) | 0.542 |

# Supplementary Table S7. (2-year lag analysis) Adjusted associations between fasting GIP and GLP-1 and any incident primary cancer in competing risk regression with 2-year lag

| **FASTING GIP** | | |
| --- | --- | --- |
|  | **SHR (CI95%)** | **p** |
| Fasting GIP | 1.05 (0.94-1.17) | 0.380 |
| Age | 0.98 (0.96-1.00) | **0.035** |
| Sex | 0.72 (0.57-0.91) | **0.006** |
| Waist circumference | 1.01 (1.00-1.02) | 0.310 |
| Diabetes | 1.12 (0.88-1.55) | 0.495 |
| Smoking | 1.55 (1.10-2.21) | **0.014** |
| Sedentary | Ref. |  |
| Moderate exercise | 1.34 (0.86-2.09) | 0.199 |
| Regular exercise | 1.64 (1.00-2.69) | 0.050 |
| Hard training | 1.01 (0.16-6.50) | 0.992 |
|  |  |  |
| **FASTING GLP1** | | |
|  |  |  |
|  |  |  |
| Fasting GLP-1 | 0.92 (0.83-1.02) | 0.097 |
| Age | 0.98 (0.96-0.99) | **0.009** |
| Sex | 0.72 (0.57-0.91) | **0.006** |
| Waist circumference | 1.01 (1.00-1.02) | 0.055 |
| Diabetes | 1.20 (0.87-1.68) | 0.270 |
| Smoking | 1.58 (1.12-2.22) | **0.008** |
| Sedentary | Reference |  |
| Moderate exercise | 1.38 (0.88-2.16) | 0.156 |
| Regular exercise | 1.61 (0.98-2.64) | 0.062 |
| Hard training | 0.97 (0.15-6.46) | 0.976 |

Values are subhazard ratios (SHR) with 95% confidence intervals (CI95%). Competing risk is death.

# Supplementary Table S8. (2-year lag analysis) Adjusted associations between post-challenge GIP and GLP-1 and any incident primary cancer in competing risk regression with 2-year lag

| **POST-CHALLENGE GIP** | | |
| --- | --- | --- |
|  | **SHR (CI95%)** | **p** |
| Post-challenge GIP | 0.93 (0.83-1.05) | 0.234 |
| Age | 0.98 (0.96-1.00) | **0.049** |
| Sex | 0.68 (0.54-0.85) | **0.001** |
| Waist circumference | 1.01 (1.00-1.02) | 0.086 |
| Smoking | 1.79 (1.25-2.57) | **0.001** |
| Sedentary | Ref. |  |
| Moderate exercise | 1.36 (0.83-2.24) | 0.222 |
| Regular exercise | 1.55 (0.89-2.69) | 0.188 |
| Hard training | 1.06 (0.17-6.84) | 0.947 |
|  |  |  |
| **POST-CHALLENGE GLP1** | | |
|  |  |  |
| Post-challenge GLP-1 | 0.90 (0.80-1.01) | 0.063 |
| Age | 0.98 (0.96-1.00) | 0.078 |
| Sex | 0.75 (0.58-0.95) | **0.019** |
| Waist circumference | 1.01 (1.00-1.02) | 0.236 |
| Smoking | 1.62 (1.14-2.31) | **0.007** |
| Sedentary | Reference. |  |
| Moderate exercise | 1.42 (0.87-2.33) | 0.161 |
| Regular exercise | 1.53 (0.88-2.64) | 0.129 |
| Hard training | 1.09 (0.17-7.14) | 0.931 |

Values are subhazard ratios (SHR) with 95% confidence intervals (CI95%). Competing risk is death.
